# A Cross-Sectional Study Assessing Undergraduate Students' Perceptions of Eating Disorders in a Dental Institution in Sangli City

**DOI:** 10.7759/cureus.81028

**Published:** 2025-03-23

**Authors:** Tanushree Dalvi, Shrivardhan Kalghatgi, Ashutosh Bhise, Rajeshree Kotawadekar, Priyanka Paul, Chetan Patil

**Affiliations:** 1 Public Health Dentistry, Bharati Vidyapeeth (Deemed to be University) Dental College and Hospital, Sangli, IND; 2 Community Medicine, Bharati Vidyapeeth (Deemed to be University) Dental College and Hospital, Sangli, IND

**Keywords:** cross-sectional studies, dentistry, eating disorders, oral health survey, student’s perception

## Abstract

Background and aim:* *Dentists and dental hygienists often lack knowledge about eating disorders (ED) and are reluctant to inform patients. This highlights the need for improved education to encourage their involvement in ED prevention and management. Hence, this study aimed to assess the knowledge, attitudes, and clinical experience related to ED among undergraduate students in a dental institute in Sangli city.

Material and methods: A descriptive, cross-sectional study was conducted among 250 third- and final-year students and interns in a dental institute in Sangli city. A self-designed questionnaire consisting of 20 questions was used to assess the knowledge, attitude, and clinical experience of students about ED. The questionnaire was tested for reliability and validity and was distributed through Google Forms (Mountain View, CA: Google LLC). SPSS (Chicago, IL: IBM Corp.) software was used for statistical analysis of data and a chi-squared test was performed.

Result: This study reveals that undergraduate dental students lacked the knowledge and experience to effectively communicate with patients who have eating disorders. Despite being familiar with the term, most participants lacked the clinical expertise to identify and guide patients with ED. The study found that 87% (n=146) of participants were familiar with the term "eating disorder" (p=0.039) with 44% (n=88) believing only hypovitaminosis was an eating disorder, 36% (n=72) believing bulimia nervosa and binge eating disorder were eating disorders (p=0.024), and 83% (n=166) believing there is a clear association between erosion and bulimia (p=0.055).

Conclusion: This study found that while most participants are aware of eating disorders, they lack clinical exposure and proper guidance, indicating the need for appropriate seminars and skill development workshops.

## Introduction

Eating disorders (ED) are biologically based psychological disorders, the physical behaviors of which can lead to severe medical problems [1]. The Diagnostic and Statistical Manual of Mental Disorders, Fifth Edition (DSM-5) currently lists the following four separate categories of eating disorders: anorexia nervosa (AN), bulimia nervosa (BN), other specified feeding and eating disorders (OSFED), binge eating disorder (BED), and unspecific feeding or eating disorders (UFED) [1,2]. It is difficult to determine the true incidence of eating disorders due to the reluctance of people affected to recognize it as a disease, thus avoiding consulting with a specialist, especially when early eating disorders are considered. In a review in 2003, only 30% of people affected by anorexia and only 6% affected by bulimia were treated at mental health centers [3].

A multidisciplinary approach, including the dental profession, is necessary when providing comprehensive care to ED patients and its prevention; the treatment of its oral manifestations can be important in the overall management of ED patients [4,5]. Early detection of ED is considered to be of utmost importance for treatment outcomes, as well as reducing the risk of somatic, psychological, and oral complications [6]. The dental team sees many patients regularly through its system of regular recalls. Thus it is possible to identify the specific signs and symptoms of ED among their patients [7,8].

Studies have shown that dentists and dental hygienists often have an insufficient level of knowledge in this regard and that it is common that they are reluctant to inform the patient/parents even if they suspect that their patients suffer from ED [9-11]. Education in this area needs to be improved, which would have the potential to encourage dentists to become more involved in both secondary prevention by identifying the early signs of ED like enamel erosion, dry mouth, and swelling of salivary glands and tertiary prevention by providing restorative and rehabilitative services, prescribing medications to increase salivation or salivary substitutes. Dentists can work alongside other healthcare providers (e.g., nutritionists, therapists, and medical doctors) to offer comprehensive care and management of ED [12]. There are no studies conducted in this regard in Sangli city. Hence, this survey aimed to investigate the knowledge, attitude, and clinical experience about eating disorders among undergraduate dental students in a dental institution in Sangli city.

## Materials and methods

The present descriptive, cross-sectional study was conducted among third-year, fourth-year, and intern undergraduate dental students in the department of public health dentistry in a dental institution in Sangli city. The period of data collection was three months from November 2023 to January 2024. Ethical approval was obtained from the institutional ethical committee of the concerned medical college in Sangli city with the reference number BV(DU)MC&H/Sangli/IEC/D-117/23. Permission to conduct the study was obtained from the head of the respective institution. Inclusion criteria consisted of students belonging to the third year, fourth year, and interns who gave voluntary written informed consent to participate in the study. Students of other years who did not give voluntary written informed consent were excluded from the study. The sample size consisted of all students fulfilling the inclusion criteria (whole sample) including 250 undergraduate dental students from the third year, fourth year, and interns.

Questionnaire details

**Figure 1:**
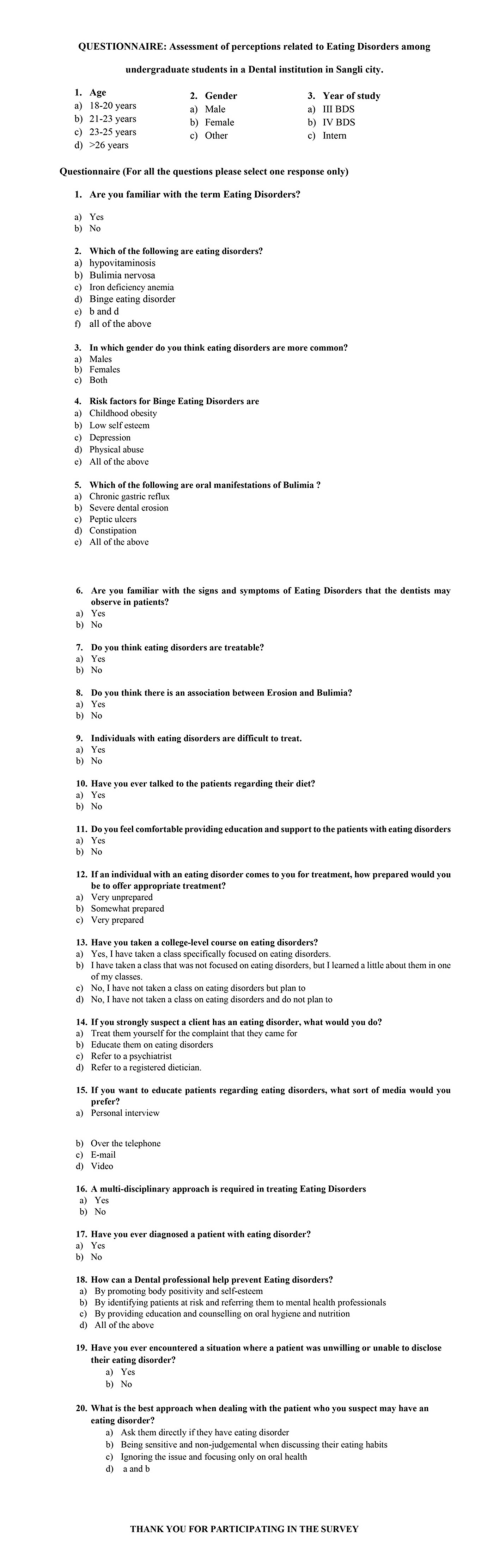
Self-designed close-ended questionnaire to assess the perceptions related to eating disorders among undergraduate students.

Figure 1 gives a detailed description of the questionnaire. Voluntary written informed consent was obtained from the study participants after explaining to them the purpose of conducting the study. The instrument for data collection was a self-designed questionnaire containing two sections. The variables assessed in section one were sociodemographic details, such as age, gender, and year of study. Section two included 20 questions to assess the knowledge, attitude, and clinical experience of undergraduate dental students related to eating disorders (ED). The questionnaire was assessed for test-retest reliability by distributing the questions to students on two different occasions within a time frame of 10 days apart and a correlation coefficient of >0.75 was obtained which indicated good reliability. Face validity was assessed considering the clarity, language of the questions, and relevance of the questions to the construct. Content validity was assessed by distributing the questionnaire to subject matter experts (SME's). Content validity index was calculated and the questions having a score of content validity index (CVI) <0.6 were modified according to the suggestions by SMEs. The questionnaire was distributed to the third- and fourth-year students and interns by the principal investigator through Google Forms (Mountain View, CA: Google LLC). The use of Google Forms eliminated interviewer bias. Students were instructed not to discuss any answers with their friends. They were also instructed to approach the investigator if they had any doubts pertaining to the questionnaire. To eliminate no-response bias, reminders were given via mail to the students who did not respond in the given time.

Statistical analysis

The data obtained were compiled systematically in an Excel sheet (Redmond, WA: Microsoft Corp.) and subjected to statistical analysis using SPSS Statistics, version 25 (Chicago, IL: IBM Corp.). Descriptive statistics were generated in terms of frequencies or percentages. Data were analyzed using chi-squared test to assess the dental student’s knowledge, attitude, and clinical experience about eating disorders. The level of significance was set at 5% and the power of the study was set at 80%.

## Results

Table 1 shows the detailed responses of the students. The total number of students who participated in the study was 250, of which 200 responses were received. The majority of the respondents belonged to the age group of 21-23 years (n=176; 88%), followed by 18-20 years (n=16; 8%), and the remaining belonged to the age group of 24-26 years (n=8; 4%). In terms of gender, 76.5% (n=153) of participants were female, and the remaining 23.5% (n=47) were male. Reports from the participants revealed that 87% (n=174) were familiar with the term 'eating disorder,' while only 13% (n=26) were unaware of the term (p=0.039). The majority of participants (n=88; 44%) thought that only "hypovitaminosis" was an eating disorder, while 36% (n=72) of participants thought both bulimia nervosa and binge eating disorder are eating disorders followed by 9% (n=18) of participants thought only binge eating disorder was an eating disorder (p=0.024). A total of 43.5% (n=87) of participants thought that eating disorders were common in both genders, while 42% (n=84) of participants thought it was more common in females, and 14.5% (n=29) participants thought it was more common in males. When asked about risk factors of binge eating disorder 72% (n=144) of participants said that all the factors (childhood obesity, low self-esteem, depression, physical abuse) were major risk factors, while 16% (n=32) of participants believed that childhood obesity was the major risk factor for binge eating disorder and 6% (n=12) suggested that low self-esteem as a risk factor.

**Table 1:** Responses by participants based on the year of study. *P<0.05 is statistically significant as analyzed by chi-squared test. III BDS: third-year bachelor of dental surgery student; IV BDS: fourth-year bachelor of dental surgery student

S. no.	Questions	III BDS	Interns	IV BDS	Total	Percentage	Pearson chi-squared value	p-Value
1	Are you familiar with the term eating disorders?						6.495	0.039*
	Yes	60	61	53	174	87.0		
	No	10	14	2	26	13.0		
2	Which of the following are eating disorders?						20.653	0.024*
	Hypovitaminosis	7	1	2	10	5.0		
	Bulimia	0	1	2	3	1.5		
	Iron deficiency anemia	7	1	1	9	4.5		
	Binge eating disorder	8	6	4	18	9.0		
	Bulimia and binge eating disorder	19	28	25	72	36.0		
	All of the above	29	38	21	88	44.0		
3	In which gender do you think eating disorders are more common?						4.731	0.316
	Males	12	9	8	29	14.5		
	Females	23	28	23	84	42.0		
	Both	35	38	34	87	43.5		
4	Risk factors for binge eating disorders are						11.110	0.196
	Childhood obesity	14	9	9	32	16.0		
	Low self-esteem	6	2	2	10	5.0		
	Depression	3	7	2	12	6.0		
	Physical abuse	2	0	0	2	1.0		
	All of the above	45	57	42	144	72.0		
5	Which of the following are oral manifestations of bulimia?						5.567	0.696
	Chronic gastric reflux	7	7	5	19	9.5		
	Severe dental erosion	15	16	11	42	21.0		
	Peptic ulcers	7	5	6	18	9.0		
	Constipation	5	1	1	7	3.5		
	All of the above	36	46	32	114	57.0		
6	Are you familiar with the signs and symptoms of eating disorders that dentists may observe in patients?						4.26	0.118
	Yes	56	51	45	152	76.0		
	No	14	24	10	48	24.0		
7	Do you think eating disorders are treatable?						1.820	0.403
	Yes	65	73	53	191	95.5		
	No	5	2	2	9	4.5		
8	Do you think there is an association between erosion and bulimia?						5.808	0.055*
	Yes	52	66	48	166	83.0		
	No	18	9	7	34	17.0		
9	Individuals with eating disorders are difficult to treat.						3.678	0.159
	Yes	31	44	32	107	53.5		
	No	30	31	23	93	46.5		
10	Have you ever talked to the patients regarding their diet?						2.17	0.33
	Yes	58	58	48	164	82.0		
	No	12	17	7	36	18.0		
11	Do you feel comfortable providing education and support to the patients with eating disorders?						3.553	0.169
	Yes	63	68	54	185	92.5		
	No	7	7	1	15	7.5		
12	If an individual with an eating disorder comes to you for treatment, how prepared would you be to offer appropriate treatment?						3.072	0.546
	Very unprepared	14	16	16	46	23.0		
	Somewhat prepared	45	43	32	120	60.0		
	Very prepared	11	16	7	34	17.0		
13	Have you taken a college-level course on eating disorders?						11.020	0.08
	Yes, I have taken a class specifically focused on eating disorders	20	15	10	45	22.5		
	I have taken a class that was not focused on eating disorders, but I learned a little about them in one of my classes	3	14	11	28	14.0		
	No, I have not taken a class on eating disorders but plan to	43	38	29	110	55.0		
	No, I have not taken a class on eating disorders and do not plan to	4	8	5	17	8.5		
14	If you strongly suspect a client has an eating disorder, what would you do?						16.371	0.012*
	Treat them yourself for the complaint that they came for	22	12	8	42	21.0		
	Educate them on eating disorders	34	51	26	111	55.5		
	Refer to a psychiatrist	3	3	4	10	5.0		
	Refer to a registered dietician	11	9	17	37	18.5		
15	If you want to educate patients regarding eating disorders, what sort of media would you prefer?						4.754	0.576
	Personal interview	48	50	42	140	70.0		
	Over the telephone	4	4	3	11	5.5		
	E-mail	5	6	0	11	5.5		
	Video	13	15	10	38	19.0		
16	A multi-disciplinary approach is required in treating eating disorders						0.033	0.983
	Yes	63	68	50	181	90.5		
	No	7	7	5	19	9.5		
17	Have you ever diagnosed a patient with an eating disorder?						2.201	0.333
	Yes	23	27	25	75	37.5		
	No	47	48	30	125	62.5		
18	How can a dental professional help prevent eating disorders?						4.093	0.571
	By promoting body positivity and self-esteem	10	9	10	29	14.5		
	By identifying patients at risk and referring them to mental health professionals	6	9	5	20	10.0		
	By providing education and counselling on oral hygiene and nutrition	12	6	9	27	13.5		
	All of the above	42	51	31	124	62.0		
19	Have you ever encountered a situation where a patient was unwilling or unable to disclose their eating disorder?						3.489	0.175
	Yes	33	33	33	99	49.5		
	No	37	42	22	101	50.5		
20	What is the best approach when dealing with a patient who you suspect may have an eating disorder?						5.029	0.540
	Ask them directly if they have an eating disorder	9	11	9	29	14.5		
	Being sensitive and non-judgemental when discussing their eating habits	20	24	21	65	32.5		
	Ignoring the issue and focusing only on oral health	5	1	2	8	4.0		
	1st and 2nd approach	36	39	23	98	49.0		

The majority of the participants (n=152; 76%) were familiar with the signs and symptoms of eating disorders. When asked about oral manifestation of bulimia, 57% (n=114) of the participants thought all the factors (chronic gastric reflux, severe dental erosion, peptic ulcer, and constipation) were associated, followed by 21% (n=42) of participants who answered severe dental erosion, and 9.5% (n=19) of participants answered chronic gastric reflux and 9% (n=18) answered peptic ulcer. Almost 95.5% (n=191) of participants thought that eating disorders were treatable. A total of 53.5% (n=107) of participants thought it was difficult to treat an individual with an eating disorder and 46.5% of (n=93) participants thought it was easy to treat a patient with an eating disorder. A total of 83% (n=166) of participants thought there was a clear association between erosion and bulimia, while 17% (n=34) participants did not believe that there was any association between erosion and bulimia (p=0.055).

From the clinical point of view, the majority of participants (n=164; 82%) talked to their patients regarding their diet, and almost 92.5% (n=185) of participants felt comfortable providing education and support to patients with eating disorders. When asked about how well prepared they were if an individual came up with an eating disorder, 60% (n=120) thought they were somewhat prepared, 23% (n=46) of participants felt they were unprepared to handle the patient, and only 17% (n=34) of participants thought that they were well prepared for such individuals. Almost 55.5% (n=111) of participants chose to educate their client or patient about eating disorders if they suspected he or she had an eating disorder, while 21% (n=42) participants chose to treat the patient for their complaints only, and 18.5% (n=37) of participants thought it was better to refer the patient to a registered dietician (p=0.012). Over 90.5% (n=181) of participants believed that it required a multidisciplinary approach to treat eating disorders. The majority of participants (n=101; 50.5%) hadn’t encountered a situation where a patient was unwilling or unable to disclose their eating disorder, while 49.5% (n=99) of participants had encountered such a situation.

A total of 55% (n=110) of participants haven’t attended any seminar regarding eating disorders but they were interested in attending one. A total of 22.5% (n=45) of participants attended the seminar on this topic, while 14% (n=28) attended a seminar that was not fully focused on eating disorders but provided some information on the topic. The remaining 8.5% (n=17) of participants stated that they were not interested in attending any seminar related to it.

A total of 70% (n=140) of the participants thought that a personal interview was the best method to educate the patient regarding eating disorders, while 19% (n=38) participants thought showing video to the patient was the best way to educate them. A total of 62.5% (n=125) of participants had not diagnosed the patient with an eating disorder and (n=75) (37.5%) of participants had diagnosed the patient with an eating disorder. Forty-nine percent (n=98) of participants thought it was best to ask directly if the patient had an eating disorder and that being sensitive and non-judgmental was the best way to approach a patient if they suspected it. Meanwhile, 32.5% (n=65) of participants thought just being sensitive and non-judgmental was the best approach to dealing with such patients and the remaining 14.5% (n=29) of participants thought asking the patient directly about their eating disorder was the best approach.

## Discussion

This study gives in-depth information regarding the knowledge, attitude, and clinical experience of undergraduate dental students regarding the ED in a dental institution in Sangli city. One of the most important findings from this research is that even after being familiar with the term ED most of the participants lacked the exact knowledge and adequate experience related to communicating with patients with ED which was in accordance with the study done by DeBate et al., where they asked volunteers about the knowledge and their clinical experience and they were unable to provide guidance or treatment to the patients [9,11].

In our study, we observed that participants thought ED equally affects both males and females but our finding was contradicted by other studies by Fairburn and Harrison and Hoek and van Hoeken, where they found that ED was more common in females [4,5]. It was clear that many patients with a diagnosis of ED do not inform the dentist (or anyone else) about their condition, because they have a huge fear of shame that was confirmed in a study by Burgard et al., where most of the participants said that fear of shame caused them to not reach out for treatment or any referral [12]. The findings from present research also showed that eating disorders need a multidisciplinary approach and there are other studies done by Aranha et al. and Kavitha et al. which found the same results [6,10].

Dentists play a critical role in the identification and prevention of eating disorders as they are often among the first healthcare professionals to identify signs of eating disorders due to the impact these conditions have on oral health (e.g., dental erosion, gingivitis, and dry mouth). Regular screenings during dental visits allow dentists to detect early signs of ED. Dentists may look for signs such as enamel erosion particularly on the inner surfaces of the upper front teeth due to gastric acid from purging behaviors. Increased cavitation can be linked to frequent purging and dietary patterns, a common symptom of bulimia or anorexia nervosa, resulting from dehydration or reduced saliva production. Once suspicious signs are identified, dentists can refer patients to mental health professionals or medical doctors specializing in eating disorders for comprehensive evaluation and treatment. Early intervention can prevent the development of severe psychological or physical complications. Dentists play an essential role in repairing dental damage caused by eating disorders, including enamel erosion, cavities, and gum disease. Restorative work such as fillings, crowns, and bonding may be necessary to restore the function and appearance of the teeth. For patients suffering from dry mouth due to eating disorders, dentists may recommend saliva substitutes or prescribe medications to promote salivation. Dentists can work alongside other healthcare providers (e.g., nutritionists, therapists, and medical doctors) to offer comprehensive care. Dentists can monitor the patient's oral health regularly, especially if they continue to struggle with an eating disorder, to address ongoing issues promptly and prevent further damage.

The present study was carried out at a single institute hence the generalizability of the findings is limited. The convenient sampling technique was used which is a type of non-probability sampling that has its limitations (under-coverage bias). On the other hand, there were certain strengths such as the response rate was 80% which was good considering it was a web-based survey, Strengthening the Reporting of Observational Studies in Epidemiology (STROBE) guidelines were followed for reporting the findings of the study, and the tool for assessment was tested for validity and reliability [13].

## Conclusions

This study concluded that most participants knew the term eating disorder but they lacked the clinical expertise, in identifying and communicating with the patients having eating disorders, so appropriate seminars and skill development workshops need to be conducted for the same. By being aware of the signs and symptoms of ED, dental students can help to identify the physical signs in patients who may be at risk such as enamel loss, tooth erosion, dry mouth, ulcers, and dentinal hypersensitivity, and refer them to mental health professionals. They can also play a key role by providing education and counseling on oral hygiene and nutrition and managing dental complications related to ED.
